# Esophageal stenosis and the Glasgow Prognostic Score as independent factors of poor prognosis for patients with locally advanced unresectable esophageal cancer treated with chemoradiotherapy (exploratory analysis of JCOG0303)

**DOI:** 10.1007/s10147-017-1154-6

**Published:** 2017-07-17

**Authors:** Tatsuya Okuno, Masashi Wakabayashi, Ken Kato, Masayuki Shinoda, Hiroshi Katayama, Hiroyasu Igaki, Yasuhiro Tsubosa, Takashi Kojima, Hiroshi Okabe, Yusuke Kimura, Tatsuyuki Kawano, Shinichi Kosugi, Yasushi Toh, Hoichi Kato, Kenichi Nakamura, Haruhiko Fukuda, Satoshi Ishikura, Nobutoshi Ando, Yuko Kitagawa, Japan Esophageal Oncology Group/Japan Clinical Oncology Group

**Affiliations:** 1 Division of Gastroenterology, Department of Internal Medicine, Graduate School of Medicine, Kobe University, 7-5-1 Kusunoki Chuo, Kobe, 650-0017 Japan; 2 Japan Clinical Oncology Group Data Center/Operations Office, National Cancer Center, Tokyo, Japan; 3 Gastrointestinal Medical Oncology Division, National Cancer Center Hospital, Tokyo, Japan; 4 Department of Gastrointestinal Surgery, Aichi Cancer Center Hospital, Nagoya, Japan; 5 Esophageal Surgery Division, National Cancer Center Hospital, Tokyo, Japan; 6 Division of Esophageal Surgery, Shizuoka Cancer Center Hospital, Shizuoka, Japan; 7 Gastrointestinal Oncology Division, National Cancer Center Hospital East, Kashiwa, Japan; 8 Department of Surgery, Kyoto University Graduate School of Medicine, Kyoto, Japan; 9 Department of Surgery, Iwate Medical University School of Medicine, Morioka, Japan; 10 Tokyo Medical and Dental University, Tokyo, Japan; 11 Department of Surgery, Niigata University Medical and Dental Hospital, Niigata, Japan; 12 Department of Gastroenterological Surgery, National Kyushu Cancer Center, Fukuoka, Japan; 13 Department of Radiology, Koshigaya Municipal Hospital, Koshigaya, Saitama Japan; 14 Department of Surgery, International Goodwill Hospital, Yokohama, Japan; 15 Department of Surgery, Keio University, School of Medicine, Tokyo, Japan

**Keywords:** T4 esophageal cancer, Chemoradiotherapy, Prognostic factor, Esophageal stenosis, Glasgow Prognostic Score

## Abstract

**Background:**

The aim of this study was to investigate the possible prognostic factors and predictive accuracy of the Glasgow Prognostic Score (GPS) for patients with unresectable locally advanced esophageal squamous cell carcinoma (LAESCC) treated with chemoradiotherapy.

**Methods:**

One hundred forty-two patients were enrolled in JCOG0303 and assigned to the standard cisplatin and 5-fluorouracil (PF)-radiotherapy (RT) group or the low-dose PF-RT group. One hundred thirty-one patients with sufficient data were included in this analysis. A Cox regression model was used to analyze the prognostic factors of patients with unresectable LAESCC treated with PF-RT. The GPS was classified based on the baseline C-reactive protein (CRP) and serum albumin levels. Patients with CRP ≤1.0 mg/dL and albumin ≥3.5 g/dL were classified as GPS0. If only CRP was increased or only albumin was decreased, the patients were classified as GPS1, and the patients with CRP >1.0 mg/dL and albumin <3.5 g/dL were classified as GPS2.

**Results:**

The patients’ backgrounds were as follows: median age (range), 62 (37–75); male/female, 119/12; ECOG PS 0/1/2, 64/65/2; and clinical stage (UICC 5th) IIB/III/IVA/IVB, 3/75/22/31. Multivariable analyses indicated only esophageal stenosis as a common factor for poor prognosis. In addition, overall survival tended to decrease according to the GPS subgroups (median survival time (months): GPS0/GPS1/GPS2 16.1/14.9/8.7).

**Conclusions:**

Esophageal stenosis was identified as a candidate stratification factor for randomized trials of unresectable LAESCC patients. Furthermore, GPS represents a prognostic factor for LAESCC patients treated with chemoradiotherapy.

**Clinical Trial Information:**

UMIN000000861.

## Introduction

Despite substantial improvements in screening and diagnosis, esophageal cancer (EC) is frequently diagnosed at a very advanced stage [1]. The thoracic esophagus lacks serosa and is closely surrounded by the trachea, bronchus, lung and aorta. Thus, esophageal cancer is more likely to invade these vital organs and become unresectable. According to the Comprehensive Registry of Esophageal Cancer in Japan, the incidence of cT4 esophageal carcinoma is approximately 15% among all EC patients [2]. Curative resection is not feasible in patients with locally advanced esophageal squamous cell carcinoma (LAESCC) if the cancer has invaded other organs, and such cases have an unfavorable prognosis [3–6]. Definitive chemoradiotherapy (CRT) is the standard of care that is currently available for unresectable locally advanced EC [5–8]. In a former phase II trial, 18 of 54 (33%) patients with clinical T4 and/or M1 lymph nodes (M1Lym) and esophageal squamous cell carcinoma (ESCC), who received cisplatin and 5-fluorouracil (PF) with concurrent 60-Gy irradiation, achieved a complete response (CR); in that study, the median overall survival (OS) and the 3-year survival rate were 9 months and 23%, respectively [7]. Additionally, a new multicenter trial JCOG0303 was conducted to evaluate the efficacy and toxicity, and particularly the long-term outcome, of CRT in patients with unresectable LAESCC. The aim of JCOG0303 was to evaluate whether RT plus low-dose cisplatin and 5-fluorouracil (LDPF) chemotherapy had an advantage in terms of survival and/or toxicity over RT plus standard-dose cisplatin and 5-fluorouracil (SDPF) chemotherapy in a randomized phase II/III trial. In a primary analysis of the phase II portion, the study was terminated due to futility. In an updated analysis, the median OS and the 3-year OS rate were 13.1 months and 25.9%, respectively, in the SDPF-RT arm, and 14.4 months and 25.7%, respectively, in the LDPF-RT arm. The primary endpoint of OS was nearly equivalent in both treatment arms [9].

The status of each patient with invasive thoracic esophageal cancer varies widely due to variability in the extent of the tumors and the nutritional and general status of the patients. Thus, establishing the factors that are involved in therapeutic effectiveness and prognosis is important for tailoring an optimized treatment strategy and further improving treatment outcomes. Emerging evidence suggests that TNM stage, a weight loss of more than 10% of one’s body mass, dysphagia, large tumors, older age, and lymphatic micrometastases (identified by immunohistochemical analysis) are independent predictors of a poor prognosis in patients with advanced EC who have undergone surgery [1]. However, little is known about the prognostic factors in patients with advanced unresectable LAESCC who have been treated with CRT. Therefore, we investigated clinical pretreatment factors that might affect the survival of patients enrolled in the JCOG0303 trial.

Moreover, hypoalbuminemia and C-reactive protein (CRP) are associated with a poor prognosis in cancer patients [10]. The Glasgow Prognostic Score (GPS) combines albumin and CRP into a risk stratification score for the prognosis of clinical outcome in cancer patients. This scoring system has been validated in colorectal cancer (CRC) and other malignant tumors, including EC [11–15]. Given that hypoalbuminemia and CRP are associated with poor prognosis in cancer patients [10], we also evaluated the potential prognostic role of the GPS on long-term outcome in patients undergoing definitive CRT for LAESCC.

## Methods

### Schema of the JCOG0303 Study

Key eligibility criteria were as follows: age of 75 years or younger, patients with histologically proven squamous cell carcinoma, adenosquamous, or basaloid carcinoma of the thoracic esophagus, and an Eastern Cooperative Oncology Group (ECOG) performance status (PS) of 0–2; in addition, patients who had any of the following conditions were included: definite clinical T4 cancer, at least 1 unresectable metastatic regional lymph node due to invasion into an adjacent organ, or computed tomography (CT)-based evidence of M1 Lym disease, such as fixed supraclavicular or celiac lymph nodes. Regional lymph nodes were defined using criteria specified by the 5th edition of the Union for International Cancer Control (UICC) TNM staging system [16]. After confirmation of eligibility, the patients were randomized at the JCOG Data Center. Written informed consent was obtained from all enrolled patients, and the institutional review boards of all participating institutions approved the study protocol. The JCOG0303 trial was registered with the UMIN Clinic Trials Registry (http://www.umin.ac.jp/ctr/) with the identification number UMIN000000861.

### Treatment

The patients were randomized to receive either SDPF-RT (arm A) or LDPF-RT (arm B). The chemotherapy regimen in arm A consisted of 70 mg⁄m^2^ cisplatin given on days 1 and 29 combined with a continuous infusion of 700 mg⁄m^2^ 5-FU given on days 1–4 and 29–32. Patients in arm B received a 1 h infusion of 4 mg⁄m^2^ cisplatin before RT, combined with a continuous infusion of 200 mg⁄m^2^ 5-FU on the first 5 days of each week. Treatment completion was defined as the termination of two courses of chemotherapy and 60 Gy of radiotherapy within 63 days.

### Statistical analysis

For the analysis of prognostic factors using pretreatment factors, the OS was calculated from the date of randomization until death from any cause, or the OS was censored at the time of the last follow-up. An initial univariable Cox regression analysis was performed to evaluate factors that could potentially affect the survival outcomes. Furthermore, a multivariable analysis that included all covariates and a multivariable analysis with the stepwise selection method were performed.

The following pretreatment factors were tested in the analyses: age (≥65 vs. <65 years), sex (male vs. female), ECOG PS (0 vs. 1 or 2), clinical T stage (T1, T2, T3 vs. T4), clinical N stage (N0 vs. N1), clinical M stage (M0 vs. M1a or M1b), histological subtype of SCC (well-differentiated or moderately differentiated vs. poorly differentiated vs. unknown), location of the primary tumor (upper vs. middle or lower), clinically diagnosed esophageal stenosis (absent vs. present), adjacent organ invasion mediated by LN metastasis (absent vs. present), intramural metastasis (absent vs. present), and the laboratory data described below, which were obtained upon enrollment in the trial. The white blood cell count, neutrophil count, hemoglobin level (Hb), CRP level, albumin level, GPT, BUN, creatinine clearance (CrC), CEA level, and SCC level were dichotomized, with cutoff points at 10,000/mm^3^, 8000/mm^3^, 13 g/dL, 1.0 mg/dL, 3.5 g/dL, 30 IU/L, 15 mg/dL, 80 mL/min, 5 ng/mL, and 1.5 ng/mL, respectively. The cutoff points of each laboratory test were determined based on a clinical perspective. Survival curves were estimated using the Kaplan–Meier method. Two-sided *p* values <0.05 were considered statistically significant. All statistical analyses were performed with SAS9.2 (SAS Institute, Cary, NC, USA).

The GPS was determined as previously described by several groups [12–15]. Patients with a CRP level ≤1.0 mg/dL and an albumin level ≥3.5 g/dL were classified as GPS0. If only the CRP level was increased or if only the albumin level was decreased, patients were classified as GPS1, and patients whose CRP level was >1.0 mg/dL and whose albumin level was <3.5 g/dL were classified as GPS2.

## Results

### Patient characteristics

**Fig. 1:**
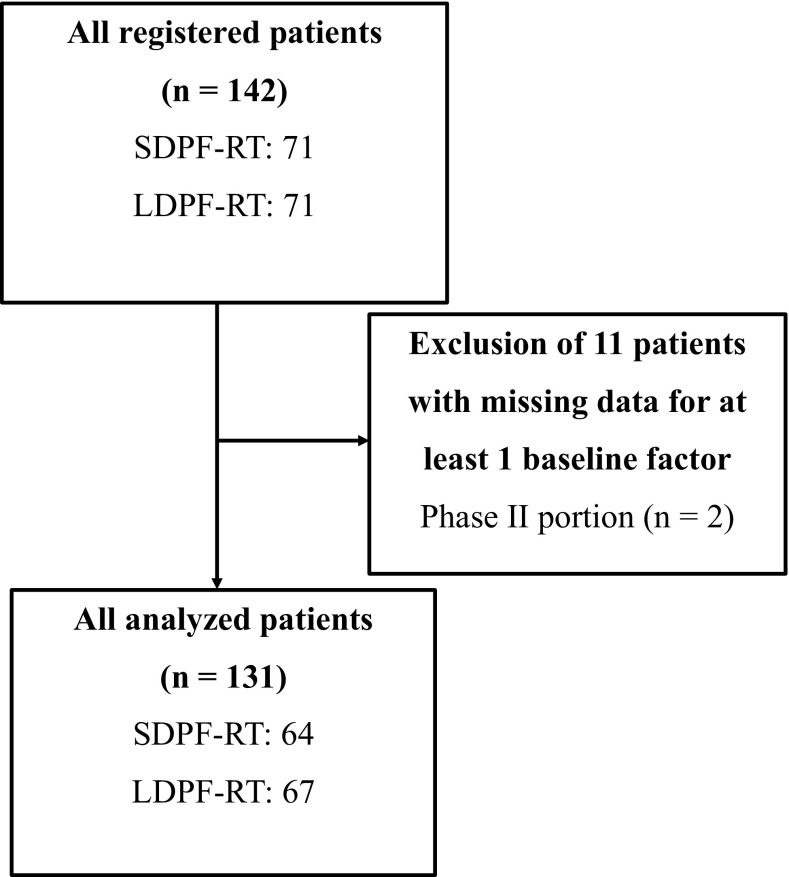
Patient flowchart

In all, 142 patients from 24 participating institutions of the Japan Esophageal Oncology Group of the JCOG were enrolled between May 2000 and May 2006. Of these, 71 patients were randomly assigned to SDPF-RT (arm A) and 71 patients were randomly assigned to LDPF-RT (arm B). Of the patients randomized into the two groups, 7 in arm A and 4 in arm B were excluded from this ancillary study because there were no documented data for the aforementioned prognostic factors. Thus, 131 patients comprised the population for the analysis based on prognostic factors (Fig. 1). The patient characteristics in each group that was subjected to the analysis using pretreatment factors are shown in Table 1.

**Table 1:** Baseline patient characteristics (*n* = 131)

Characteristic	SDPF-RT (*n* = 64)	LDPF-RT (*n* = 67)
Gender		
Male	61	58
Female	3	9
Age (years)		
Median	63	62
Range	37–75	43–74
ECOG PS		
0	30	34
1	33	32
2	1	1
cTNM (UICC 5th edition)		
T1	2	0
T2	5	2
T3	9	12
T4	48	53
N0	9	9
N1	55	58
M0	38	40
M1a	8	14
M1b	18	13
Clinical stage (UICC)		
IIb	2	1
III	36	39
IVa	8	14
IVb	18	13

*ECOG PS* Eastern Cooperative Oncology Group performance status

**Fig. 2:**
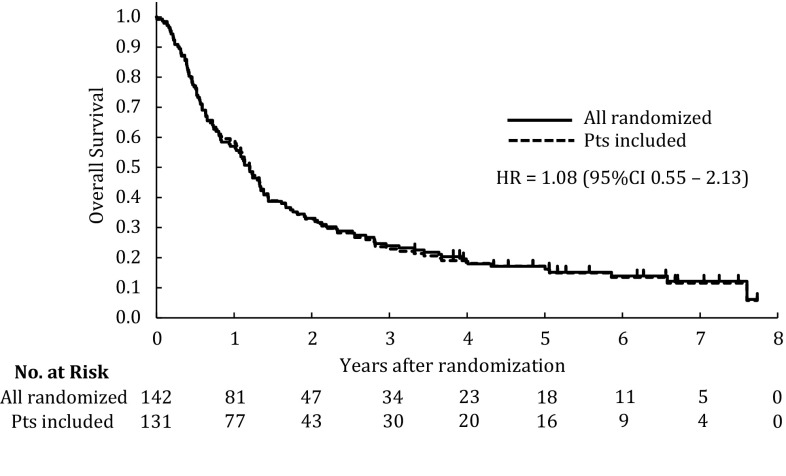
Kaplan–Meier estimates of the overall survival of all randomized (*n* = 142) patients, including the study (*n* = 131) patients

The median overall survival times (MSTs) of all randomized 142 patients and of the final 131 patients included in this study were 14.4 months. Among all randomized patients, the 1- and 3-year survival rates were 57.0 and 23.9%, respectively, while those rates of the patients included in this study were 58.8 and 22.9%, respectively (Fig. 2).

### Multivariable analysis

**Fig. 3:**
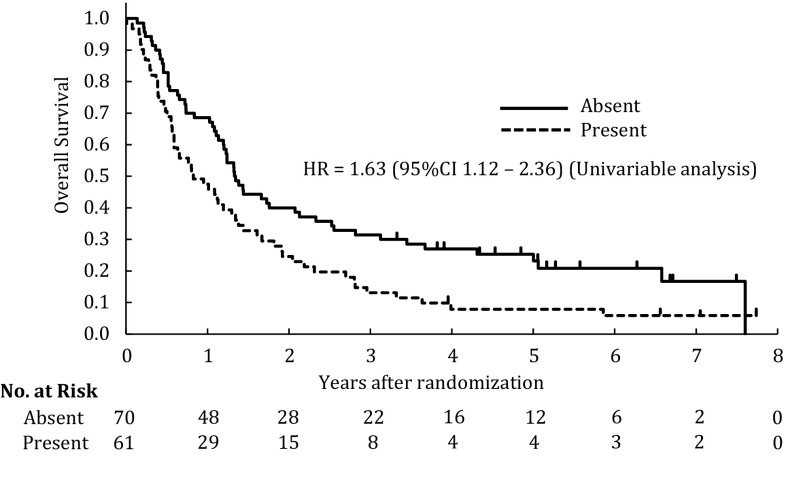
Kaplan–Meier estimates of overall survival in the absence of stenosis (*n* = 70) and in the presence of stenosis (*n* = 61)

A multivariable analysis that included all covariates demonstrated that esophageal stenosis, lower Hb, clinical M1 stage, location of the primary tumor (middle or lower) and histological subtype of SCC (unknown) were significantly associated with poor survival. A multivariable analysis with stepwise selection also confirmed esophageal stenosis as an indicator of poor survival, although a serum Alb level <3.5 g/dL was not an independent prognostic factor in the multivariable analysis that included all covariates. The hazard ratio for patients with esophageal stenosis (present) was 1.60 (95% CI 1.10–2.32) compared with patients without esophageal stenosis (absent) (*p* = 0.014) in the multivariable analysis with stepwise selection (Table 2). Patients with esophageal stenosis had a median survival of 9.9 months, while patients without esophageal stenosis had a median survival of 16.0 months (*p* = 0.001) (Fig. 3).

**Table 2:** Multivariable Cox regression analysis of overall survival according to pretreatment factors

Factors	Levels	*N*	Univariable analysis		Multivariable analysis			
					Including all covariates		Stepwise selection	
			HR (95% CI)	*p* value	HR (95% CI)	*p* value	HR (95% CI)	*p* value
Age	65>	85	1	0.320	1	0.231		
	65≤	46	1.219 (0.825–1.802)		1.335 (0.832–2.140)			
Sex	Female	12	1	0.464	1	0.744		
	Male	119	0.784 (0.409–1.504)		0.881 (0.414–1.878)			
PS	0	64	1	0.446	1	0.360		
	1 or 2	67	1.156 (0.796–1.678)		1.228 (0.791–1.908)			
T-stage	T4	101	1	0.558	1	0.177		
	T1, T2, T3	30	0.873 (0.555–1.373)		0.646 (0.343–1.217)			
N-stage	N0	18	1	0.348	1	0.976		
	N1	113	1.308 (0.747–2.292)		1.011 (0.512–1.996)			
M-stage	M0	78	1	0.070	1	0.002		
	M1a or M1b	53	1.426 (0.972–2.092)		2.271 (1.368–3.772)			
Location of the primary tumor	Ut	37	1	0.396	1	0.009		
	Mt or Lt	94	1.199 (0.788–1.825)		2.068 (1.198–3.569)			
Histological subtype of SCC	Well or Moderately differentiated	97	1	All (0.372)	1	All (0.024)		
	Poorly differentiated	19	1.138 (0.680–1.903)	0.623	1.311 (0.741–2.320)	0.352		
	Unknown	15	1.494 (0.844–2.644)	0.168	2.844 (1.332–6.070)	0.007		
Stenosis	Absent	70	1	0.010	1	0.002	1	0.014
	Present	61	1.629 (1.123–2.361)		2.014 (1.293–3.137)		1.598 (1.101–2.319)	
Adjacent organ Invasion mediated by LN metastasis	Absent	105	1	0.822	1	0.545		
	Present	26	1.056 (0.657–1.698)		1.193 (0.675–2.108)			
Intramural metastasis	Absent	107	1	All (0.081)	1	All (0.216)		
	Present	13	1.641 (0.913–2.948)	0.098	1.883 (0.927–3.824)	0.080		
	Unknown	11	1.735 (0.922–3.266)	0.088	1.057 (0.456–2.451)	0.897		
WBC	10,000≥	113	1	0.611	1	0.095		
	10000<	18	0.868 (0.503–1.498)		0.449 (0.176–1.148)			
ANC	8000≥	121	1	0.920	1	0.533		
	8000<	10	1.036 (0.520–2.065)		1.477 (0.434–5.027)			
Hb	13≥	85	1	0.042	1	0.008		
	13<	46	0.664 (0.447–0.986)		0.509 (0.310–0.836)			
CRP	1.0 (mg/dL)≥	69	1	0.117	1	0.367		
	1.0 (mg/dL)<	62	1.344 (0.929–1.945)		1.266 (0.758–2.114)			
Alb	3.5 (g/dL)>	40	1	0.018	1	0.988	1	0.025
	3.5 (g/dL)≤	91	0.621 (0.420–0.921)		0.996 (0.559–1.773)		0.636(0.429–0.944)	
GPT	30>	105	1	0.032	1	0.134		
	30≤	26	1.639 (1.044–2.573)		1.531 (0.878–2.670)			
BUN	15.0>	98	1	0.863	1	0.336		
	15.0≤	33	0.963 (0.628–1.478)		1.269 (0.781–2.063)			
CrC	80.0>	65	1	0.405	1	0.077		
	80.0≤	66	1.171 (0.807–1.698)		1.500 (0.957–2.352)			
CEA	5>	110	1	0.849	1	0.733		
	5≤	21	0.954 (0.587–1.550)		0.902 (0.501–1.626)			
SCC	1.5≥	67	1	0.265	1	0.622		
	1.5<	64	1.236 (0.852–1.793)		1.115 (0.723–1.721)			

*HR* hazard ratio

### GPS as an independent prognostic factor for survival

**Fig. 4:**
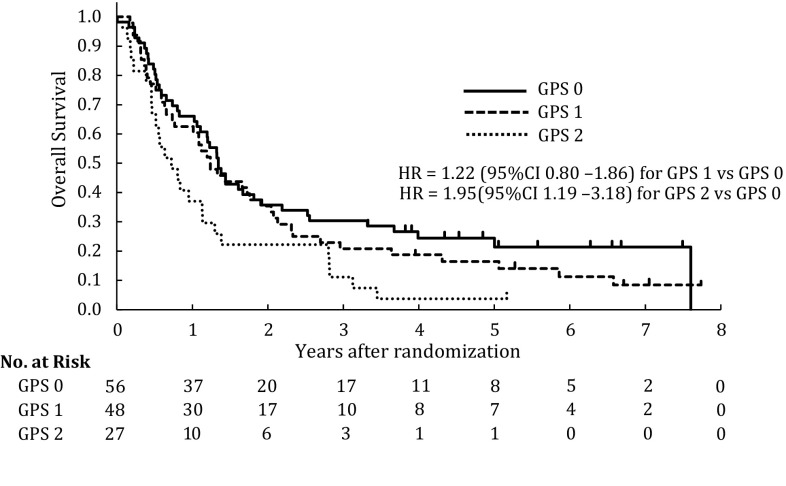
Kaplan–Meier estimates of overall survival in GPS0 (*n* = 56), GPS1 (*n* = 48) and GPS2 (*n* = 27) patients

The association between survival and the GPS in patients with esophageal carcinoma is shown in Fig. 4. The HR for overall survival in GPS1 patients to GPS0 patients was 1.22 (95% CI 0.80–1.86; *p* = 0.35) and was 1.95 (95% CI 1.19–3.18; *p* = 0.008) in GPS2 patients to GPS0 patients. Patients with a GPS of 0 had a median survival of 16.1 months, while patients with a GPS of 1 and 2 had a median survival of 14.9 and 8.7 months, respectively (*p* = 0.001) (Fig. 4). These findings suggest that an elevated GPS is associated with poor prognosis. Moreover, we confirmed that the presence or absence of stenosis and the GPS score did not have remarkable relationship.

## Discussion

Here, we retrospectively investigated the therapeutic outcomes of patients who were prospectively registered in the JCOG0303 trial and evaluated pretreatment clinical parameters that might consistently affect survival. In the present study, two multivariable analyses revealed esophageal stenosis as the only single factor that was associated with poor prognosis in patients with unresectable LAESCC. To our knowledge, this is the first study to report an association between treatment outcome and clinically diagnosed esophageal stenosis.

Currently, the established factors of a poor prognosis are PS, tumor size and TNM stage, but in our analysis, no correlations were found between these factors and prognosis. This might reflect the fact that our patients in the present study were limited to those with T4 and/or unresectable supraclavicular lymph node metastasis (i.e., those at a relatively advanced stage with a poor prognosis). Zenda et al. described that low pretreatment levels of Hb were significantly associated with the efficacy of CRT in patients with T4/M1 EC [17]. Similarly, low levels of Hb have been reported to be associated with sensitivity to chemoradiotherapy [18], locoregional control [19, 20], and survival [17, 19, 21, 22] after radiation therapy for EC with or without chemotherapy. In the present study, the pretreatment Hb level did not demonstrate clinical significance in terms of its association with survival, which may have been because included patients were limited to those who had met the eligibility criteria of the prospective study.

Thus far, SCC and CEA are well-known tumor markers for esophageal cancer [23]. Nevertheless, their significance as prognostic factors is controversial. In this study, using univariable and multivariable analyses, the relationship between survival and the levels of CEA and SCC was examined; however, they were not found to be prognostic factors.

Patients with advanced EC comprise a heterogeneous population, as represented by differences in variables such as age, co-morbidities, primary tumor location, and ECOG PS. Additionally, simple and inexpensive markers are desirable to establish a prognosis of ESCC. Therefore, we evaluated the utility of the GPS in unresectable LAESCC because these markers can be easily analyzed using typical blood tests. The GPS, which takes into account the serum CRP and albumin levels, simply reflects the systemic inflammation status of patients with cancer and their prognosis, as described for various cancer types. In addition, a series of work by McMillan and other groups outlined the indisputable association between an increased CRP level and poor survival in various tumor types [10, 11, 24–29]. The present study shows that the pretreatment GPS was an independent prognostic factor in patients with unresectable LAESCC. These findings suggest the pivotal role of systemic inflammation in patients with EC who undergo CRT for LAESCC. Most reports regarding GPS for ESCC investigated patients who underwent esophagectomy and described the GPS as reflecting deeper tumor involvement, higher numbers of nodal metastases, and more advanced cancer stage [13, 30, 31]. These findings suggest that the GPS of patients with unresectable LAESCC might represent a synthetic marker for the prediction of overall survival because it reflects both tumor factors and patient background. Recently, in a retrospective study at a single institution, Ohira et al. reported that GPS1/2 scores were closely associated with poor prognosis compared with patients with a GPS0 score with the same cancer stage as those in our study [32].

This analysis does have some limitations. One of the limitations is that the patients enrolled in this clinical trial, who might have a better prognosis, are compared with patients in clinical practice. Therefore, other factors that affect the prognosis of “real” patients may be obscured. Another limitation is the definition of “stenosis”. In the JCOG0303 trial, stenosis of the primary lesion was not strictly defined, and the clinical investigators who participated in this study diagnosed it subjectively. Thus, “stenosis” seemed to vary among investigators. Some investigators reported stenosis in patients who experienced dysphagia, while other investigators reported stenosis in patients whose primary lesions were too narrow for an endoscopy to be performed. Collecting and evaluating data in an objective manner using the dysphagia score (DS) would be a better procedure to follow in the next clinical trial [33].

In conclusion, esophageal stenosis was thought to be a potential stratification factor in a randomized trial that included patients with unresectable LAESCC. Furthermore, the GPS represents a prognostic factor in patients with LAESCC who are treated with CRT. These data can be easily collected before treatment. Further studies are needed to clarify the association between these pretreatment data and clinical outcomes and to be able to use these parameters as stratification factors in the next prospective randomized trial of CRT for LAESCC.
